# Improved Large-Scale Homology Search by Two-Step Seed Search Using Multiple Reduced Amino Acid Alphabets

**DOI:** 10.3390/genes12091455

**Published:** 2021-09-21

**Authors:** Kazuki Takabatake, Kazuki Izawa, Motohiro Akikawa, Keisuke Yanagisawa, Masahito Ohue, Yutaka Akiyama

**Affiliations:** Department of Computer Science, School of Computing, Tokyo Institute of Technology, Tokyo 152-8550, Japan; takabatake@bi.c.titech.ac.jp (K.T.); izawa@bi.c.titech.ac.jp (K.I.); akikawa@bi.c.titech.ac.jp (M.A.); yanagisawa@c.titech.ac.jp (K.Y.); ohue@c.titech.ac.jp (M.O.)

**Keywords:** homology search, genome sequence, metagenomic analysis, reduced amino acid

## Abstract

Metagenomic analysis, a technique used to comprehensively analyze microorganisms present in the environment, requires performing high-precision homology searches on large amounts of sequencing data, the size of which has increased dramatically with the development of next-generation sequencing. NCBI BLAST is the most widely used software for performing homology searches, but its speed is insufficient for the throughput of current DNA sequencers. In this paper, we propose a new, high-performance homology search algorithm that employs a two-step seed search strategy using multiple reduced amino acid alphabets to identify highly similar subsequences. Additionally, we evaluated the validity of the proposed method against several existing tools. Our method was faster than any other existing program for ≤120,000 queries, while DIAMOND, an existing tool, was the fastest method for >120,000 queries.

## 1. Introduction

Metagenomics is the study of microorganism genomes in the environment, such as the soil, ocean, and living organisms, achieved by extracting and sequencing DNA. This method can reveal the existence and ratio of microorganisms in a specific clade within an environment, and also provide genomic information of unknown and uncultured microorganisms.

The DNA sequences of most microorganisms in the environment are unknown; thus, metagenomic analysis refers to the genomic information of not only the same species, but also that of closely related organisms. A homology search must be performed against enormous databases to identify sequences that are similar to those obtained by DNA sequencing. Since changes in genomic information occur at the amino acid level, a six-frame translation of the DNA sequence is performed prior to the homology search, focusing only on the coding region. While DNA sequences are represented by four letters (A, T, G, and C), protein sequences are represented by 20 standard amino acid letters. Further, while comparisons between DNA sequences often distinguish only two states of each base (match or mismatch), similarities between protein sequences are based on a substitution matrix that represents the likelihood of each amino acid being substituted with another amino acid [1]. Therefore, performing a homology search on protein sequences is more challenging than on DNA sequences.

The Smith–Waterman algorithm [2] is the most rigorous method to obtain optimal sequence alignment by means of dynamic programming during a homology search. This algorithm is implemented in SSEARCH [3] and other software; however, SSEARCH is too slow to search against a large number of sequences within a realistic execution time. For this reason, programs that perform fast homology searches are now widely used, such as BLAST [4,5]. Nevertheless, the amount of data has drastically increased with the advent of high-throughput next-generation DNA sequencers. For example, the Illumina NovaSeq6000 DNA sequencer can output up to 6T bases in a single run of a few days, requiring hundreds of thousands of CPU days for BLAST to process that volume of data. Thus, faster homology search programs such as RAPSearch2 [6], GHOSTZ [7], and DIAMOND [8] have been proposed. While there is a tradeoff between search speed and accuracy, further acceleration while maintaining high accuracy is required for comprehensive metagenomic analyses.

In general, homology search tools identify subsequences (seeds) that have a high degree of similarity between database and query sequences, thereby substantially reducing the number of candidate sequences in the database. This is called a seed search strategy. RAPSearch2, GHOSTZ, and DIAMOND achieve highly efficient and accurate searches by employing reduced amino acid alphabets in their seed search strategies. These reduced amino acid alphabets cluster the 20 standard amino acids according to the scores between them, yielding an alphabet with a smaller number of representative characters. The reduced amino acid alphabets proposed by Murphy et al. [9] are commonly used in existing methods. Performing a seed search based on a sequence converted to a reduced amino acid alphabet enables treating similar amino acids, such as Asp and Glu, as the same character. As a result, database sequences similar to the query sequence can be quickly detected by exact-match search. Existing methods have used single patterns of reduced alphabets. However, the amino acids that constitute the functional sites of proteins are strongly conserved, whereas the peripheral amino acids that maintain the structure are loosely conserved. Therefore, we hypothesized that significant seed hits could be identified more rapidly by searching for exact matches in the center of the seed using a less compressed amino acid alphabet, while searching at both ends of the seed using a more compressed amino acid alphabet.

In this study, we proposed a new seed search algorithm that employs multiple reduced amino acid alphabets with different numbers of characters to search for similar subsequences in a two-step seed search (TSSS) strategy, with the aim of implementing a faster but accurate homology search program. The implementation is open-sourced at https://github.com/akiyamalab/tsss (accessed on 17 September 2021) under the MIT license.

## 2. Methods

### 2.1. Reduced Amino Acid Alphabets

**Figure 1:**
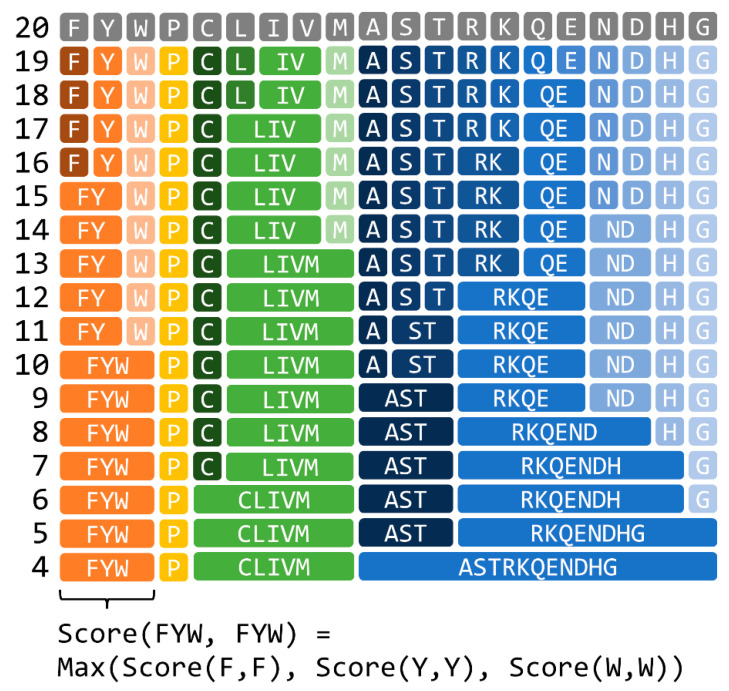
Reduced amino acid alphabet generated using the method proposed by Murphy et al. [9].

A reduced amino acid character represents a cluster of standard amino acids, as shown in Figure 1. Treating a match of amino acids represented by the same reduced amino acid alphabet as a perfect match provides a fast relaxed search that allows the substitution of an amino acid with a similar amino acid. In this study, we used the reduced amino acid alphabets generated using the method proposed by Murphy et al., based on the BLOSUM62 substitution matrix, which describes the similarity score between each of the 20 standard amino acids. First, correlation coefficients were calculated for all pairs of amino acids based on the BLOSUM62, and then the amino acids were grouped by hierarchical clustering. The size of the reduced amino acid alphabet was user-defined, ranging from 4 to 18 characters in the current study. The maximum score obtained by a perfect match of any standard amino acid in the group was used to define the match score for a matching reduced amino acid alphabet character in an identical group, as an example is shown in the bottom part of Figure 1.

### 2.2. Two-Step Seed Search (TSSS)

**Figure 2:**
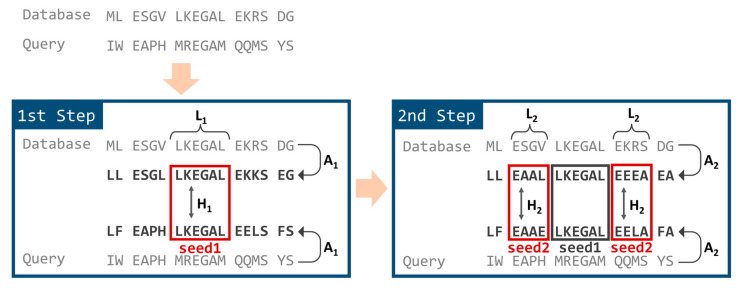
Seed search steps of TSSS.

In TSSS, the seed search was divided into two steps employing different reduced amino acid alphabets, as shown in Figure 2. In the first step, the database and query sequences were converted into the first reduced amino acid alphabet, A_1_. Subsequences starting from all possible positions of the query sequence were enumerated (referred to as *seed1*) with a residue length of L_1_. Then, matching portions were searched against the database where *seed1* matched, with a Hamming distance ≤ H_1_.

In the second step, the *seed1* hits obtained in the first step were extended in both directions with a residue length of L_2_. Each extended area at both ends was named *seed2*. The *seed2* subsequences were converted into the second reduced amino acid alphabet, A_2_, and Hamming distances between the query and database sequences were calculated individually for both *seed2* areas. If the Hamming distances of both *seed2* areas were less than or equal to threshold H_2_, the whole region (*seed1* plus both *seed2* areas) was treated as a final seed hit for the following alignment calculation.

In the case of H_1_ > 0 (or H_2_ > 0), we generated all words within the designated Hamming distance for the query seed and then searched database positions that exactly matched with one of the words.

Larger values of A_1_, A_2_, L_1_, or L_2_ corresponded with smaller numbers of obtained seed hits for the alignment step, which resulted in faster processing but degraded sensitivity. Similarly, smaller values of H_1_ or H_2_ resulted in faster and less sensitive processing. Therefore, the combination of values greatly affected the specifications of TSSS, necessitating optimization of these parameters.

There is a possible alternative searching strategy where the *seed1* plus both *seed2* areas are at first searched with a small (loose) reduced amino acid alphabet, and then candidates are narrowed down by searching the *seed1* area with a large (tight) reduced amino acid alphabet. Nevertheless, this approach might not be so efficient with the current TSSS procedure because enumerating all patterns within a Hamming distance requires high computational cost in the first step for a longer seed.

### 2.3. TSSS Procedure

**Figure 3:**
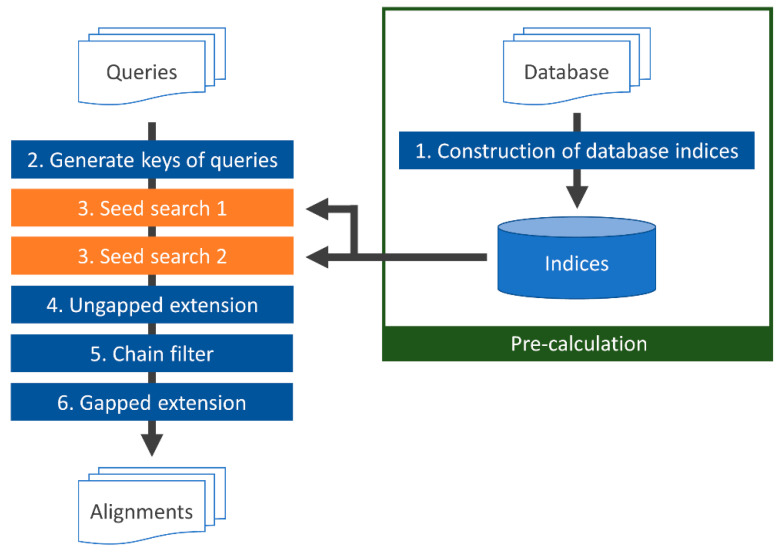
TSSS flowchart.

Figure 3 displays a flowchart of TSSS. First, (1) indices of the *seed1* and *seed2* subsequences in the database were constructed with the corresponding reduced amino acid alphabets A_1_ and A_2_. Once the homology search was initiated, (2) the DNA query sequence was translated into a protein sequence, followed by conversion of the translated sequence to both the first and second reduced amino acid alphabets A_1_ and A_2_. Then, seeds of the query were enumerated and translated with the same reduced amino acid alphabets. Next, (3) the seed was searched against the database to find positions (seed hits) with high similarity between the database and query sequences. Finally, (4–6) the identified seeds were extended (ungapped and gapped seed extensions) with alignment, and the score was calculated. The details of each process are described in the following sections.

#### 2.3.1. Building a Database Subsequence Index (1)

First, all protein sequences in the database were concatenated by inserting a predetermined delimiter character such as ‘#’. All subsequences with the lengths of the two seeds, *seed1* L_1_ and *seed2* L_2_, were obtained by repeatedly shifting one character at a time in the concatenated sequence. Subsequences containing the delimiter were discarded. Then, depending on parameters A_1_, A_2_, L_1_, and L_2_, the *seed1* and *seed2* areas were enumerated and indices were generated that mapped each subsequence to a position in the database.

#### 2.3.2. Generating Keys for Query (2) and Searching for Seeds (3)

DNA sequence reads obtained by a DNA sequencer were translated into six frames and concatenated using the delimiter. The same operation described in Section 2.3.1 was performed on the concatenated query to enumerate keys of the subsequence. Keys containing the termination codon were discarded.

In the seed search, parameters H_1_ and H_2_ (described in Section 2.2) were used to search for database positions of the seed hits. An identified seed hit was recorded as a candidate for alignment, and the next operation was executed.

#### 2.3.3. Ungapped Extension (4) and Chain Filtering (5)

In the extension step, an ungapped extension procedure was performed around the seed hit to further narrow down significant database positions. Although the seed search used reduced amino acid alphabets, the process after the ungapped extension step calculated an alignment score including the seed region based on the 20 standard amino acids. Termination codons were not discarded in the ungapped and gapped extension procedures, and were regarded as a single virtual amino acid. The similarity score between a termination codon and each amino acid was defined by the BLOSUM62 matrix. In the ungapped extension step, as in BLAST, X-dropoff [4] was used to terminate the extension when the score decrease from the peak value was greater than the value determined by the score matrix and user-defined parameter. Only subsequences with scores exceeding the threshold were recorded as candidates for the next gapped extension step.

Where a long region in the database matched the query, many subsequences had almost the same results in the gapped extension. For this reason, TSSS used chain filtering to eliminate redundant gapped extension trials by combining subsequences located in neighboring positions into one subsequence [5]. Subsequences were combined into one longer subsequence when subsequences of the ungapped extension overlapped, or when the ungapped extension score between two subsequences was greater than or equal to the threshold.

#### 2.3.4. Gapped Extension (6)

The gapped extension in TSSS used the same method as BLAST. As in the ungapped extension, we employed X-dropoff to terminate the extension when the score decrease from the peak value was greater than the threshold. We also used a Gotoh algorithm [10] that considered affine gaps, which reduced the penalty for consecutive gaps in alignment.

### 2.4. Evaluation Procedure

#### 2.4.1. Computing Environment and Comparison Programs

We evaluated the computational speed and accuracy of TSSS using the f_node of the TSUBAME3.0 supercomputer at Tokyo Institute of Technology. This computing environment consisted of two Intel Xeon E5-2680 v4 (14 cores, 2.4 GHz) CPUs and 256 GB of memory. We used GCC (version 4.8.5) as the compiler, with the optimization option -O3.

BLAST [4,5] (version 2.7.1), RAPSearch2 [6] (version 2.22), GHOSTZ [7] (version 1.0.2), and DIAMOND [8] (version 0.9.14.115) were used as comparison programs. The following options for all programs were employed: BLOSUM62 for the substitution matrix, no SEG filter to ignore low-complexity regions of sequences, and 10 alignments per query. Each program supported multi-thread operations, but was executed with a single thread unless otherwise noted. The detailed options for each program are presented in Table 1.

The sizes of the reduced amino acid alphabets used in the comparison programs are listed in Table 2. Although most alphabets were 10 characters in size, the seed search algorithms of each program differed, making a simple comparison difficult. For example, DIAMOND used multiple patterns of spaced seeds that do not necessarily require an exact match of contiguous subsequences.

#### 2.4.2. Datasets

We employed the KEGG GENES prokaryotes database (acquired in February 2019) [11,12] as the protein sequence database. This database consists of approximately 17.7 million protein sequences, with a total residue length of approximately 5.6 billion residues. SRR5788325 [13], a set of DNA sequences obtained from the NCBI Sequence Read Archive, was used as the query data. The DNA sequences were quality controlled using PRINSEQ-lite [14] (version 0.20.4) prior to the experiments.

#### 2.4.3. Calculation of Accuracy

To evaluate the search accuracy of each program, the optimal hits with the Smith–Waterman algorithm were needed for each query sequence. We used SSEARCH with the E-value threshold of 10^−5^ to obtain them. Reference hits used in the evaluation consisted of up to 10 optimal hits, and thus, the maximum number of reference hits was 10 for each query. Then, the top 10 hits of each homology search program were retained, and the obtained hits resulting in the same sequences as the reference hits were counted as matches. The accuracy of the search program was measured as the ratio of the number of matches to the number of reference hits (≤10). The E-value of the alignment was based on the output value of each program, although a previous study suggests that for a more precise calculation, the E-value should consider frameshift alignment [15].

## 3. Results and Discussion

### 3.1. TSSS Performance with Various Parameters

We evaluated all 1344 combinations of TSSS parameters listed in Table 3. The values were manually selected from the appropriate ranges for each parameter. As for the sizes of the reduced amino acid alphabets A_1_ and A_2_, we expected TSSS to be effective when A_1_ > A_2_. However, we also examined TSSS performance when the same-sized reduced amino acid alphabet was used for the entire seed (A_1_ = A_2_), and when a larger reduced amino acid alphabet was used at both ends of the seed compared with that used in the center of the seed (A_1_ < A_2_).

**Figure 4:**
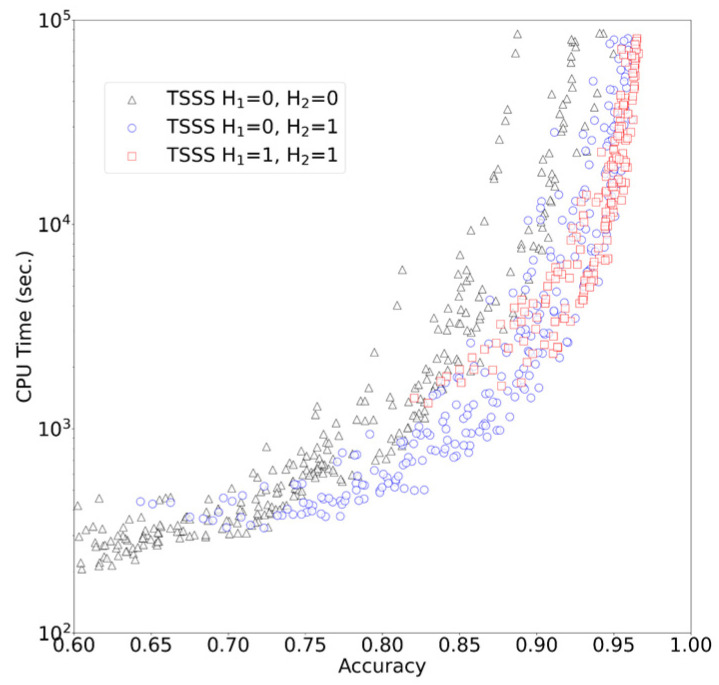
TSSS results.

The results of TSSS with 1344 different parameter sets (with 50,000 queries) are presented in Figure 4. A unique Pareto surface existed for each combination of H_1_ and H_2_. When (H_1_, H_2_) = (0, 0), the program searched only for seeds that exactly matched in total length. In the range where the accuracy ≤ 0.7, this parameter set resulted in shorter CPU times than other parameter sets, and the search was performed at a higher speed rather than improved accuracy. When (H_1_, H_2_) = (1, 1), the search speed was improved in the range where accuracy ≥ 0.95, resulting in one of the best parameter sets that emphasized accuracy. This parameter set allowed up to three Hamming distances for the entire seed, and the program was able to search for seeds with high similarity while allowing mismatches. When (H_1_, H_2_) = (0, 1), speed and accuracy were balanced. In particular, this parameter set outperformed other parameter sets with an accuracy range of 0.7 to 0.9.

Figure S1 in the Supplementary Materials illustrates the results of TSSS for the magnitude relationship between A_1_ and A_2_. The settings of A_1_ = A_2_, the same settings as the one-step seed search, is not the worst among all settings; however, the figure revealed the Pareto surfaces were formed by the use of a less compressed amino acid alphabet in the center of the seed while a more compressed amino acid alphabet at both ends of the seed (A_1_ > A_2_). This result was consistent with the assertion that TSSS can efficiently reduce the number of alignment candidates by applying strict filtering in the center of the seed and relatively loose filtering in the periphery of the seed.

Further observations revealed that long seeds with a small reduced amino acid alphabet tended to yield worse results because the conversion to a reduced amino acid alphabet with fewer characters made it difficult to obtain a seed with high similarity. The distribution of accuracy and computing time for all combinations of A_1_ and A_2_ are shown in Figures S2 and S3.

### 3.2. Comparison with Existing Tools

**Figure 5:**
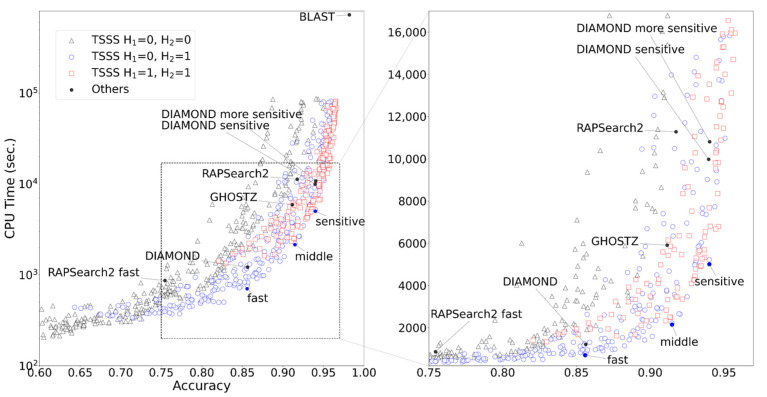
Accuracy and CPU time for each method.

Comparing TSSS against existing tools, BLAST demonstrated the highest accuracy but the longest execution time, while DIAMOND-sensitive and DIAMOND-more sensitive were the next most accurate tools (Figure 5). The results indicated that some parameter sets in TSSS yielded faster results than existing tools with equivalent accuracy. The parameter sets that yielded the same accuracy as DIAMOND, GHOSTZ, and DIAMOND-sensitive and had the shortest CPU times were named TSSS fast, middle, and sensitive. The parameter details for the three TSSS methods are presented in Table 4.

**Figure 6:**
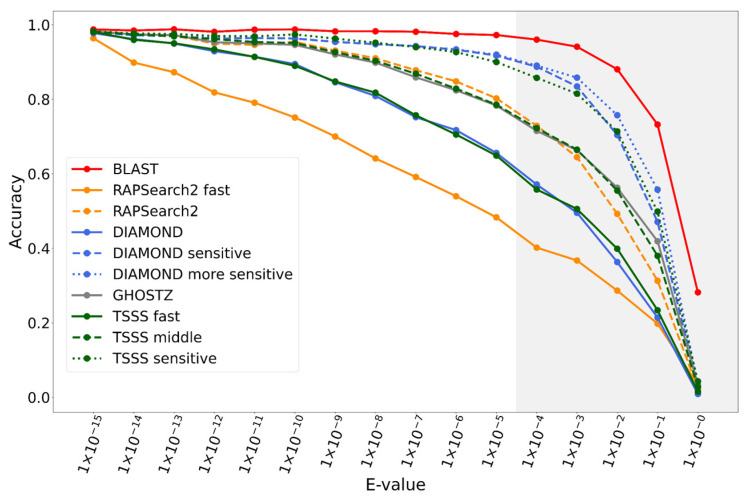
Accuracy of each method according to E-value.

Additionally, we also evaluated the change in accuracy for varying E-value thresholds by SSEARCH when creating the reference hits. The strict E-value threshold (e.g., 10^−15^) evaluates the search accuracy of alignments with high identity, whereas a loose E-value threshold evaluates the search accuracy of alignments likely containing many mismatches and gaps. Figure 6 illustrates the accuracy order of the methods was almost consistent with the E-values. Our TSSS fast, middle, and sensitive methods were comparable with those of the target competitors, DIAMOND, GHOSTZ, and DIAMOND-sensitive, over a wide E-value range.

### 3.3. Speed Comparison by Query Size

**Figure 7:**
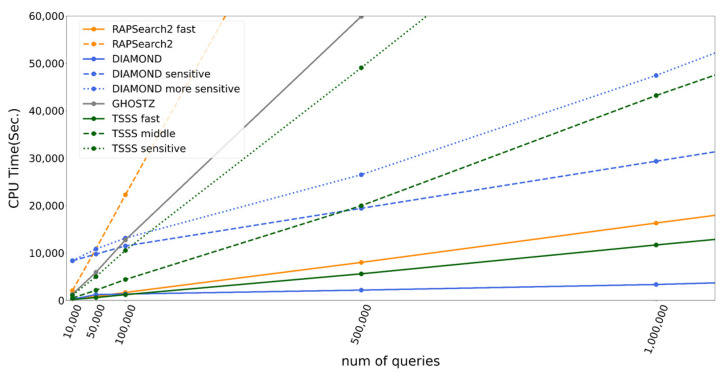
CPU time according to number of queries for each program.

The number of queries greatly affects the calculation time; thus, we tested TSSS and existing tools with various query sizes. Figure 7 presents the execution times and their linear extrapolations since calculation time was almost directly proportional to the number of queries. Interestingly, DIAMOND demonstrated different behavior from the other programs. This is because the time DIAMOND takes to construct the data structure for the seed search appeared as the intercept, which then accelerated the calculation per query time, reflected as the slope inclination. The results demonstrated that the TSSS sensitive method was faster than DIAMOND-sensitive at the same accuracy when the number of queries was small, whereas DIAMOND-sensitive had an advantage when the number of queries was large. DIAMOND-sensitive became faster than the TSSS sensitive method when the number of queries > 120,000 with a length of 150 bases.

To compare the execution times in detail, we excluded estimated preparation overhead and calculated the execution time per query in reference to BLAST, as shown in Table 5. According to the table, DIAMOND was up to 4.9 times faster than the TSSS fast method. Similarly, DIAMOND-sensitive and DIAMOND-more sensitive were up to 4.9 and 2.2 times faster than the TSSS sensitive method, respectively. However, the TSSS middle method was faster than RAPSearch2 and GHOSTZ, which had similar accuracies. The TSSS middle method was 336.1, 5.0, and 2.2 times faster than BLAST, RAPSearch2, and GHOSTZ, respectively. Interestingly, TSSS was faster than GHOSTZ even though GHOSTZ accelerates computation by effectively managing redundancy in the database, which is not performed by TSSS. These results may indicate that our TSSS strategy is superior to the traditional one-step seed search algorithm.

### 3.4. Parallel Efficiency

**Figure 8:**
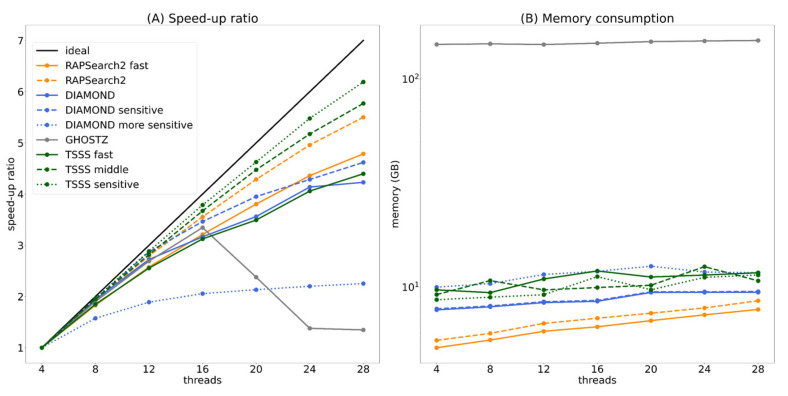
Speed-up ratio (**A**) and memory consumption (**B**) for parallel execution.

As described in Section 2.4.1, TSSS and existing tools support multi-thread operation. Figure 8 presents the strong scaling performance and memory consumption when each program was executed parallelly for one million query sequences. Furthermore, each program was executed with 4, 8, 12, 16, 20, 24, and 28 threads, and the speed-up ratio was calculated based on the CPU time when executed with four threads.

In the speed-up ratio graph (Figure 8A), DIAMOND and the TSSS fast method overlapped and displayed similar parallel efficiency. The TSSS middle method demonstrated better parallel efficiency than both RAPSearch2 and GHOSTZ. Furthermore, the TSSS sensitive method had higher parallel efficiency than DIAMOND-sensitive and DIAMOND-more sensitive. Note that the parallel efficiency of GHOSTZ was significantly decreased when the number of threads was ≥20, which suggests that the seed search was not suitable for parallelization or there may be a problem in the implementation of parallelization.

In the memory consumption graph (Figure 8B), all programs, excluding GHOSTZ, was approximately 10 GB with 28 threads, which is acceptable when using large-scale computing resources such as supercomputers. In contrast, GHOSTZ had a large memory consumption of approximately 150 GB with 28 threads, which may limit the execution of this program depending on the computing environment.

### 3.5. Removal of Low-Complexity Regions

The low-complexity regions (LCR) should be carefully removed in sequence homology search. Several filtering approaches have been eagerly studied, including hard masking, soft masking, and gentle masking [16]. Such an LCR filter is generally used in the preprocessing of database and query sequences. Though many homology search software have built-in SEG filter options, previous assessments turned off the filtering option because it sometimes results in missing important homologous sequence and may affect performance comparison [7,17].

In this study, we did not use built-in SEG filter as described in Section 2.4.1. However, our quality control procedure described in Section 2.4.2 was not perfect and allowed some LCR sequences to remain both in the database and query sequences. We investigated and found that the database sequences that appeared in the top 10 reference hits included LCR in their whole protein sequence with a modest frequency. Less than about 5% of the reference hit entries (24,575 of 487,344) had an LCR on their whole sequence. Thus, the incomplete LCR-filtration may have had moderate but non-ignorable influence on the results of accuracy assessment. We assume that the influence was not significantly biased for specific tools. However, it is desirable to achieve more accurate assessment with the use of a sophisticated LCR-filtration algorithm.

## 4. Conclusions

Herein, we proposed a homology search algorithm that employed TSSS with different reduced amino acid alphabets and compared the implementation of this method against several existing homology search tools. TSSS was 5.0 and 2.2 times faster than RAPSearch2 and GHOSTZ, respectively, using parameters providing the same accuracy. In addition, the same parameters achieved 336 times faster search than that with BLAST. These results indicate that the TSSS’s two-step seed search strategy is more effective than the traditional one-step seed search strategy widely used among homology search tools such as DIAMOND and GHOSTZ. However, the implementation of TSSS has scope for improvement in terms of the pre-calculation of the seed search. DIAMOND was faster than TSSS when the number of queries was sufficiently large and up to 4.9 times faster using the parameter set providing the same accuracy. The most important reason for its faster speed is the double indexing technique that DIAMOND uses to construct a data structure for all query sequences as well as for all database sequences, thereby shortening the execution time per query. Therefore, double indexing or the construction of a suitable data structure specialized for TSSS must accelerate our implementation. Furthermore, TSSS combined with the spaced seed technique utilized in DIAMOND instead of enumeration of all words will enhance the performance. The clustering of database sequences technique utilized in GHOSTZ is another option to enhance the performance.

## Figures and Tables

**Table 1:** Options for comparison programs.

Program	Options
BLAST	-outfmt 6 -comp_based_stats 0 -seg no
RAPSearch2 fast	-b 0 -t n -a t
RAPSearch2	-b 0 -t n
GHOSTZ	-q d -F
DIAMOND	-f 6 -e 10 -p 1 --masking 0 --comp-based-stats 0
DIAMOND-sensitive	-f 6 -e 10 -p 1 --sensitive --masking 0 --comp-based-stats 0
DIAMOND-more sensitive	-f 6 -e 10 -p 1 --more-sensitive --masking 0 --comp-based-stats 0

**Table 2:** Reduced amino acid alphabets used in comparison programs.

Program	Size of Reduced Amino Acid Alphabet
RAPSearch2	10
GHOSTZ	10
DIAMOND	11

**Table 3:** TSSS parameter ranges.

Parameter	Description	Range
(H_1_, H_2_)	Hamming distances allowed for *seed1* and *seed2*	{(0, 0), (0, 1), (1, 1)}
L_1_	Length of *seed1*	{2, 4, 6, 8}
A_1_	Size of reduced amino acid alphabet for *seed1*	{6, 8, 10, 12, 14, 16, 18}
L_2_	Length of *seed2*	{2, 3, 4, 5}
A_2_	Size of reduced amino acid alphabet for *seed2*	{4, 6, 8, 10}

**Table 4:** Parameter details of representative TSSS methods.

Name	H_1_	H_2_	L_1_	A_1_	L_2_	A_2_
Fast	0	1	4	18	5	6
Middle	0	1	4	16	4	8
Sensitive	0	1	2	18	5	8

**Table 5:** Execution speed ratio against NCBI BLAST.

Program	Speed Ratio
BLAST	1.0
RAPSearch2 fast	890.2
DIAMOND	6122.7
TSSS fast	1241.9
RAPSearch2	65.2
GHOSTZ	121.2
TSSS middle	336.1
DIAMOND-sensitive	731.4
DIAMOND-more sensitive	347.0
TSSS sensitive	148.0

## Data Availability

The query DNA sequences used in this study are available in SRR5788325 obtained from the NCBI Sequence Read Archive. The KEGG GENES prokaryotes database were downloaded in February 2019 under the KEGG FTP academic subscription license.

## Supplementary Materials

The following are available online at https://www.mdpi.com/article/10.3390/genes12091455/s1, Figure S1: Results of TSSS for magnitude relationship between A_1_ and A_2_, Figure S2: Boxplots based on accuracy for all combinations of A_1_ and A_2_, Figure S3: Boxplots based on CPU time for all combinations of A_1_ and A_2_.
