# Supplementary material for: Improved Large-Scale Homology Search by Two-Step Seed Search Using Multiple Reduced Amino Acid Alphabets
Source: Genes (Basel). 2021 Sep 21;12(9):1455. doi: 10.3390/genes12091455 (PMC8469100; doi:10.3390/genes12091455)
Supplement: Supplementary file 1 [file genes-12-01455-s001.zip › genes-1325410-supplementary.pdf]

Supplementary Materials

Improved Large-scale Homology Search by Two-Step Seed Search using Multiple Reduced Amino Acid Alphabets

Kazuki Takabatake, Kazuki Izawa, Motohiro Akikawa, Keisuke Yanagisawa, Masahito Ohue, and Yutaka Akiyama*

Department of Computer Science, School of Computing, Tokyo Institute of Technology; takabatake@bi.c.titech.ac.jp (K.T.); izawa@bi.c.titech.ac.jp (K.I.); akikawa@bi.c.titech.ac.jp (M.A.); yanagisawa@c.titech.ac.jp (K.Y.); ohue@c.titech.ac.jp (M.O.)
*Correspondence: akiyama@c.titech.ac.jp

Supplementary Figures

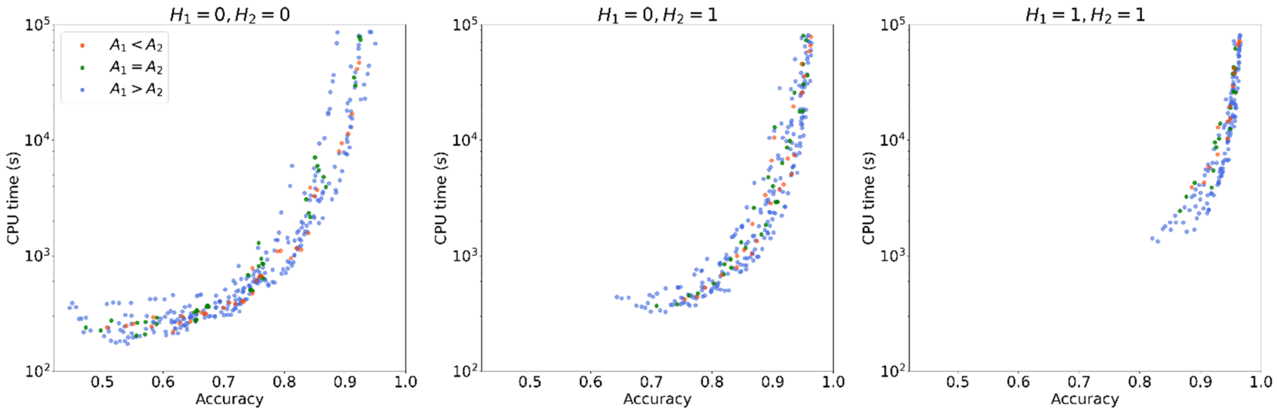

Figure S1. Results of TSSS for magnitude relationship between A_1 and A_2.

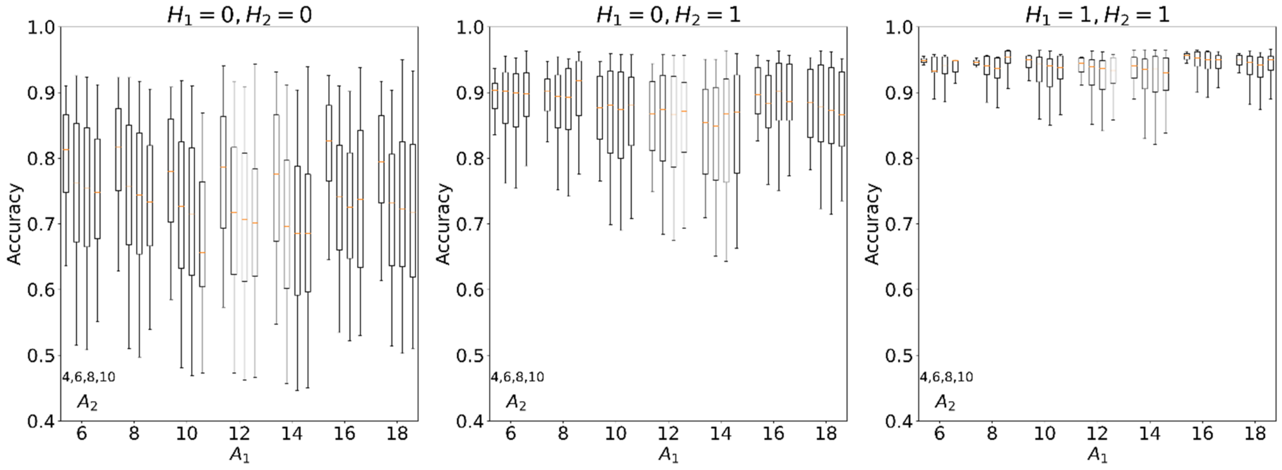

Figure S2. Boxplots based on accuracy for all combinations of A_1 and A_2.

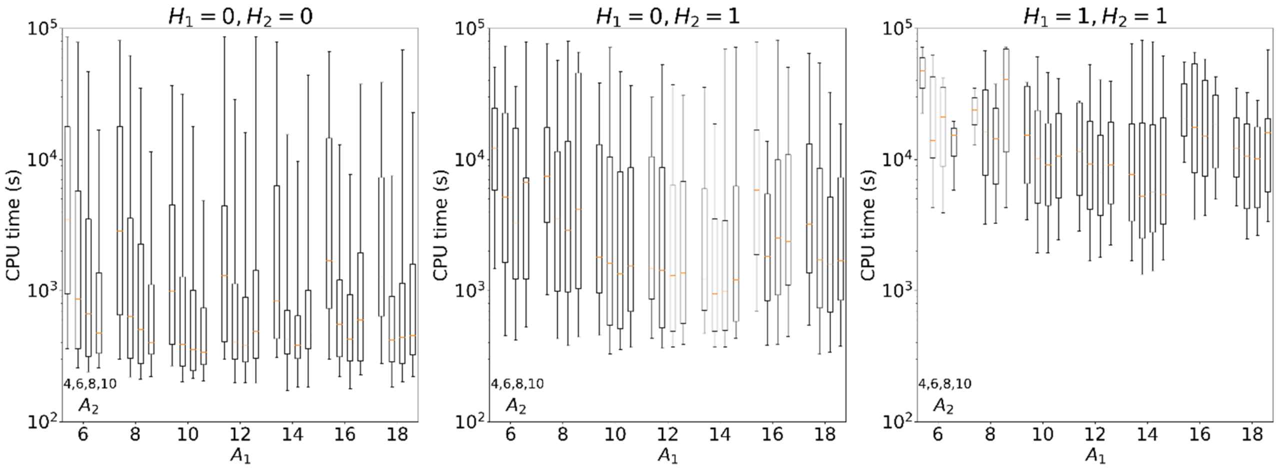

Figure S3. Boxplots based on CPU time for all combinations of A_1 and A_2.
